# Recommendations for online learning challenges in nursing education during the COVID-19 pandemic

**DOI:** 10.4102/curationis.v45i1.2360

**Published:** 2022-10-27

**Authors:** Boitumelo J. Molato, Leepile A. Sehularo

**Affiliations:** 1 Department of Nursing, Faculty of Health Sciences, North-West University, Mahikeng, South Africa

**Keywords:** recommendations, online learning, challenges, nursing education, COVID-19, pandemic

## Abstract

**Background:**

Nursing education institutions have had to change from face-to-face to online learning because of the coronavirus disease 2019 (COVID-19) pandemic. The online learning mode, however, had several challenges.

**Objectives:**

To explore and describe recommendations made to address the online learning challenges in nursing education during the COVID-19 pandemic.

**Method:**

This study adopted a narrative literature review to achieve its objectives. The search for the relevant literature used Google Scholar, ScienceDirect, African Journal (previously SAePublications), EBSCOhost, EBSCO Discovery Service and Scopus databases.

**Results:**

There were four findings identified from the literature search: provision of adequate resources, monitoring of academic dishonesty, provision of technical support and revision of the curriculum.

**Conclusion:**

More work in nursing education is necessary to address the challenges of adopting online learning during and after the COVID-19 pandemic. To meet the issues of online learning in nursing education, thorough preparations and safeguards are necessary.

**Contribution:**

The outcomes of this study will benefit nursing education by incorporating recommendations from many studies to overcome online learning issues in nursing education during the COVID-19 pandemic.

## Introduction

The coronavirus disease 2019 (COVID-19) is a contagious disease that originated in China and spread to other nations worldwide. The World Health Organization’s (WHO) Emergency Committee, directed by the Director General, declared COVID-19 an epidemic of a public health emergency of international concern (PHEIC) on 30 January 2020, based on its features (WHO 2020). The declaration of COVID-19 as a PHEIC did not endure long, as the label changed to pandemic quickly. The decision to declare it a pandemic stemmed from the rapid rise in the number of countries affected (WHO 2020). The COVID-19 pandemic has resulted in a high fatality rate and poses a serious public health threat globally (ILO, FAO, IFAD & WHO 2020). Furthermore, according to the WHO, the COVID-19 pandemic has had a detrimental influence not only on public health but also on the worldwide education community because of preventive steps to flatten the infection curve (Giovannella 2021:2). In reaction to the COVID-19 pandemic, colleges and universities immediately switched from face-to-face to online learning mode to save the academic year. The departments and schools that started online learning included nursing education institutions (NEIs). The COVID-19 pandemic has had a significant impact on the educational system. Nurse educators and student nurses were undecided about whether online learning would meet the programme’s objectives (Sasmal & Roy 2021:1892). Web-based software was primarily used in this teaching and learning method to direct, produce and deliver learning content, as well as to promote a two-way contact between students and nurse educators (Mukhtar et al. 2020:27). The advantage of online learning is that it is simple to manage and instructors and students can access it anywhere, regardless of the distance between them (Mukhtar et al. 2020:29). Students also learn to be self-directed learners, which is a valuable skill for supporting lifelong learning in health care professionals and boosting confidence (Kaur & Sharma 2020:497). Students now have quicker and easier access to learning packages which include recordings of class activities. Furthermore, students are inspired to actively participate in the learning process (Almahasees, Mohsen & Amin 2021:7).

Regardless of the advantages of online learning during the COVID-19 pandemic, student nurses experienced obstacles (Goodwin et al. 2022:7). Literature reveals students were using phones to access online learning because of a lack of other resources, such as laptops. Under normal conditions, phones are not suitable for online learning (Bester et al. 2021:5); some nursing students were obliged to go to an Internet cafe to complete assignments and homework, and access to a stable Internet connection was also an issue (Bester et al. 2021:5).

A study by Salmani, Bagheri and Dadgari (2022:7) found that the students were cheating on online exams and distributing assignments between themselves. Furthermore, when writing summative evaluations, most of the students referred to textbooks and learning resources or divided the course materials among their peers, with each person accountable for studying and understanding one piece. The computer illiterate student nurses received inadequate support from the NEIs, which resulted in them having difficulty in accessing online learning (Thapa, Bhandari, Pathak & 2021:13). Anecdotally, nursing is a profession that incorporates both theoretical and practical elements to improve programme outcomes, but it was difficult for nurse educators to teach the clinical component through online platforms during COVID-19 pandemic. As a result, most of the student nurses were impacted by this intervention, as they were required by various statutory organisations to amass a certain number of hours in the clinical setting (Michel et al. 2020:904). As the patient admissions were confined to emergencies and COVID-19 cases because of the pandemic’s surrounding events, the integration of theory into practice was hampered, and nurses in practice were unable to show skills, coach or supervise student nurses because of insecurity (Ulenaers et al. 2021:4). The insecurities that nurses had were that they were afraid that they might contact the virus when demonstrating skills to the students. It is obvious that the COVID-19 pandemic had an impact on the nursing education community.

During the pandemic, however, there was little information about the recommendations for online learning challenges in nursing education. A complete grasp of the recommendations for online learning issues in nursing education during the COVID-19 pandemic, as reported in numerous researches, is required to aid nurse educators and other stakeholders in implementing appropriate measures to overcome the constraints (Langegård et al. 2021:9). The researchers considered it necessary to conduct a narrative literature review on the recommendations to address online learning challenges in nursing education during the COVID-19 pandemic to attain this stated goal. The recommendations section of one study report contained key ideas for the best course of action in a specific situation (Hasa 2018); in other words, this part served as a helpful guide for resolving certain challenges and achieving a positive end. The recommendation section suggests specific activities to address issues raised in the research, as well as future research projects (Hasa 2018). Therefore, the review of literature is imperative to extract recommendations suggested by the conducted studies to address the challenges faced in nursing education during the COVID-19 pandemic. The proposed narrative literature review was guided by the following question: what are the recommendations to address the online learning challenges in nursing education during the COVID-19 pandemic?

## Aim and objectives

This study aimed to explore and describe the recommendations to address online learning challenges in nursing education during the COVID-19 pandemic.

## Design and methods

**FIGURE 1:**
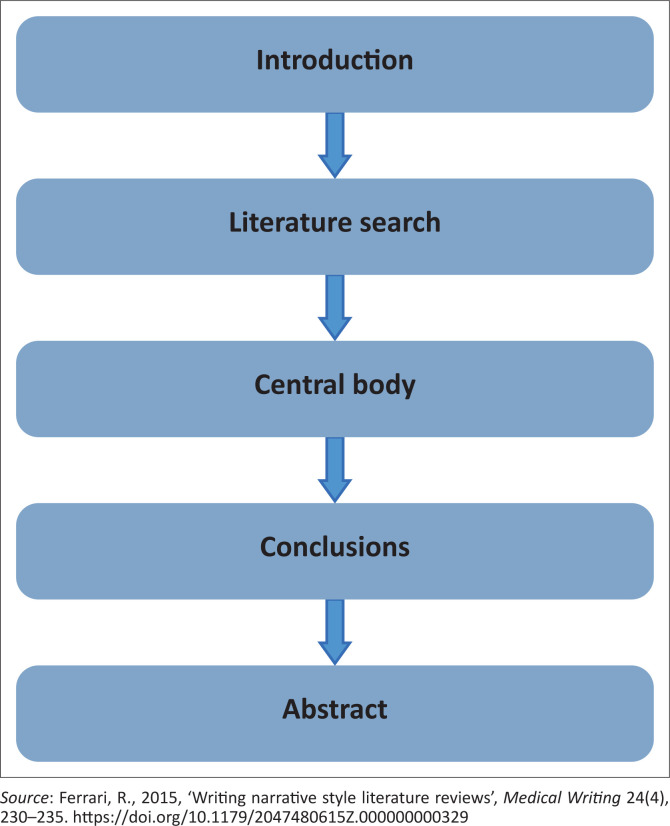
Narrative literature review steps as suggested by Ferrari (2015).

This manuscript used a narrative literature review design and methods to explore and describe the literature on recommendations to address online learning challenges in nursing education during the COVID-19 pandemic. The following steps, as suggested by Ferrari (2015:232), guided this narrative literature review: introduction, literature search, central body, discussion and abstract. Figure 1 shows the steps of narrative literature review used in this study.

### Step 1: Introduction

The first step of a narrative literature review, according to Ferrari (2015:232), is the introduction. The introduction appears in page 1 of this article. This section of the introduction was to provide a brief overview of the phenomenon discussed in this research. The study topic led the entire process of describing the problem that will be solved (Ferrari 2015:232). The introduction defines the context of the research by summarising current knowledge and information on the background of the topic, as well as stating the objective of the study (Ferrari 2015:232). The study’s purpose and research objectives are simply the objectives the researchers aim to attain.

### Step 2: Literature search

The second step of a narrative literature review is literature search (Ferrari 2015:232). The following databases were searched for literature relating to the topic of this study: Google Scholar, African Journal (previously SAePublications), ScienceDirect, EBSCOhost, EBSCO Discovery Service (EDS) and Scopus. This narrative literature review was limited to studies published between 2020 and 2022. The keywords used to search for data were ‘recommendations’, ‘online learning’, ‘challenges’, ‘nursing education’ and ‘COVID-19 pandemic’. Additionally, there were abstracts scrutinised to ensure that they addressed the goals of this research. This research also considered articles written in English. There was due acknowledgement of authors of the publications utilised in this narrative literature review, with a reference list created to show all articles considered for this study (Ferrari 2015:232). See Table 1 for databases used to search the literature.

**TABLE 1:** Literature search.

Databases	Search terms	Inclusion criteria	Exclusion criteria
African Journal (previously SAePublications) EBSCO Delivery Service (EDS) EBSCOhost Google Scholar ScienceDirect Scopus	Recommendations Online learning Challenges Nursing education COVID-19 pandemic	English studies focusing on recommendations to address online learning challenges in nursing education during the COVID-19 pandemic	Newspapers Conference reports Other databases not mentioned in this study

EDS, EBSCO Delivery Service.

For verification of the studies selected, see Table 2 for more details.

**TABLE 2:** Verify the availability of all the selected studies.

Author(s) and year	Aim	Design and sample	Rigour
Bester et al. (2021)	To explore the barriers and enablers for ICT adoption by a diverse group of student nurses in a private nursing education institution in Free State province	Qualitative, explorative, interpretive-descriptive design, 17 student nurses	Aims and objectives clearly stated Study design adequately described Results consistent Implications discussed Quality appraisal = high quality (A)
Oducado (2021)	Determine the expectations of and readiness for online learning of sophomore nursing students in one nursing school in a developing country	Cross-sectional research design, 149 student nurses	Aims and objectives clearly stated Study design adequately described Results consistent Implications discussed Quality appraisal = high quality (A)
Salmani et al. (2022)	Describe Iranian nursing students’ experiences of e-learning during the COVID-19 pandemic	Qualitative design; student nurses who had successfully completed the 4th, 6th and 8th semester of the undergraduate nursing programme were recruited to the study	Aims and objectives clearly stated Study design adequately described Results consistent Implications discussed Quality appraisal = high quality (A)
Thapa et al. (2021)	Identify the nursing students’ attitude towards the practice of e-learning amid COVID-19	Web-based cross-sectional study was conducted among nursing students with a sample size of 470	Aims and objectives clearly stated Study design adequately described Results consistent Implications discussed Quality appraisal = high quality (A)
Ullah et al. (2021)	Identify the challenges faced by students during online learning Identify the difference between male and female students regarding challenges faced during online learning Determine the effectiveness of online learning in Pakistan from students’ perspectives	Descriptive research design, 550 students	Aims and objectives clearly stated Study design adequately described Results consistent Implications discussed Quality appraisal = high quality (A)
Buthelezi and Van Wyk (2020)	To explore students’ perceptions of e-learning, their perceived challenges with technology on a compulsory postgraduate nursing module and associations between demographic data and listed challenges	Exploratory quantitative study, 60 student nurses	Aims and objectives clearly stated Study design adequately described Results consistent Implications discussed Quality appraisal = high quality (A)
Bdair (2021)	To explore the nursing students’ and faculty members’ perspectives of online learning during the COVID-19 era	Qualitative study using a descriptive-phenomenology method; a sample of 10 student nurses and 10 faculty members were invited to participate	Aims and objectives clearly stated Study design adequately described Results consistent Implications discussed Quality appraisal = high quality (A)
Tolyat et al. (2022)	To explain the experiences of nursing education amid the COVID-19 pandemic	Qualitative content analysis approach; 12 nurse educators and 7 student nurses	Aims and objectives clearly stated Study design adequately described Results consistent Implications discussed Quality appraisal = high quality (A)
Oducado and Estoque (2021)	To determine the undergraduate nursing students’ stress, satisfaction and academic performance during online learning	108 second-year undergraduate student nurses	Aims and objectives clearly stated Study design adequately described Results consistent Implications discussed Quality appraisal = high quality (A)

COVID-19, coronavirus disease 2019; ICT, information communication technology.

### Step 3: Central body

A narrative literature review’s third step is the central body. According to Ferrari (2015:232), this step includes the findings of the literature review, thoroughly explained and cited, with the sources pertinent to the setting of the narrative literature review. In this narrative literature review, the researchers utilised their voices to describe the results in accordance with the title (Ferrari 2015:232).

### Step 4: Conclusions

The fourth step of a narrative literature review is conclusions (Ferrari 2015:232). The conclusion section highlights and discusses the main points identified in this narrative literature review (Ferrari 2015:232).

### Step 5: Abstract

The last step of a narrative literature review is writing the abstract (Ferrari 2015:232). In line with Ferrari (2015:232), the findings of this study are addressed and summarised in accordance with the journal guidelines. The abstract of this study, follows the guidelines of the *Curationis* journal, which is the journal of the Democratic Nursing Organisation of South Africa, accredited by the Department of Higher Education and Training.

## Ethical considerations

Because of the nature of this study, which is a narrative literature review, there was no ethical approval required to examine the current literature on recommendations for addressing online learning challenges in nursing education during the COVID-19 pandemic (Sehularo et al. 2021:3). The process for finding articles was detailed in the section on design and methods. The authors of the articles cited in this study have due acknowledgement in the text and the reference list (Kara 2019). Each author made a unique contribution to the creation of this work (Sehularo et al. 2021:3).

## Search strategy

**FIGURE 2:**
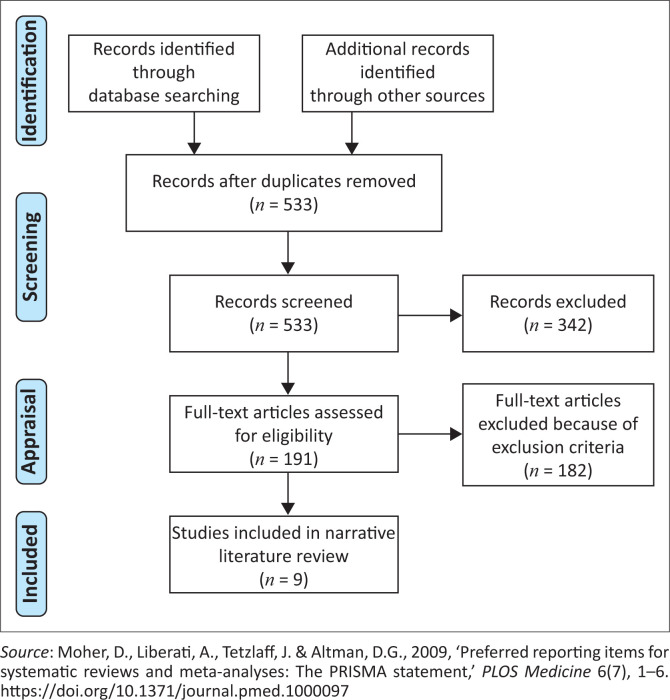
Flow chart of the search strategy (adapted from the 2009 PRISMA flow diagram).

For more details regarding search strategy for the articles used in this study, see Figure 2.

## Results and discussion

The goal of this narrative literature review was to explore and describe the recommendations to address online learning challenges in nursing education during the COVID-19 pandemic. This study revealed four findings that answered the review question, namely provision of adequate resources, monitoring of plagiarism, provision of technical support and the revision of the curriculum. The supply of adequate resources was the first finding discovered by this narrative literature review, which means that institutions of higher learning should ensure student nurses receive equipment so that they may conveniently access online learning. Other researchers, such as Oducado (2021:1175), recommended that higher education institutions should guarantee that student nurses have appropriate resources, such as laptops, tablets, computers, data or Wi-Fi, to enable them to utilise the online learning mode fully. This study’s findings are consistent with those of a qualitative, exploratory, interpretive-descriptive study conducted in the Free State province of South Africa. According to this study’s findings, student nurses should be provided with computers and other devices with a solid network to use when they are not on campus (Bester et al. 2021:8). This project will benefit marginalised students who cannot afford to buy the necessary devices to access online learning.

The second finding identified is the necessity to monitor academic dishonesty. Academic dishonesty is academic behaviour that goes against set assessment criteria and other institutional norms, such as when students engage in ways that provide them an unfair advantage in their evaluations (Saana et al. 2016:1). The study conducted by Head et al. (2022:53) recommended that all NEIs should implement software to track academic dishonesty among student nurses. The objective of the software is to instil academic integrity ideals in students. Academic integrity, according to Gamage, Silva and Gunawardhana (2020:5), entails being honest in the academic work at university, being fair to others and taking responsibility for learning.

The provision of technical support is the third finding found by this study. Three sources mentioned the provision of technical support as one of the recommendations for addressing the challenges in nursing education during the COVID-19 pandemic (Bdair 2021; Buthelezi & Van Wyk 2020; Thapa et al. 2021:13). This study recommends that NEIs equip students and nurse educators with the technical skills required for online learning. The conclusions of this study are in accordance with those of a qualitative study conducted in Namibia, which recommended that nurse educators receive ongoing training on platforms utilised to enhance online learning (Shindjabuluka, Ashipala & Likando 2022:6). Users must be computer literate and have the necessary abilities and expertise to participate in online teaching and learning. Consequently, NEIs should implement infrastructure to support online teaching and learning; this includes comprehensive workshops for nurse educators and nursing students on how to conduct online learning, utilising digital platforms. Furthermore, there should be emotional and psychological assistance provided to those primarily involved in the online learning process.

The fourth finding revealed was revision of the curriculum. It was difficult to teach the clinical component through online platforms during the COVID-19 pandemic. In this regard, change in the learning environment, integrating technology into education, is no longer an option but a need for all authorities that requires flexibility, creativity and innovation (Tolyat, Vagharseyyedin & Nakhaei 2022:45). This study strongly suggests redesigning the curriculum so that there can be delivery of theoretical and clinical components through online platforms (Oducado & Estoque 2021:149). For both theoretical and practical learning, the nursing curriculum should be converted from traditional to online. Similarly, an exploratory convergent mixed methods study conducted in Norway found that during the COVID-19 pandemic, the NEIs altered the curriculum to address clinical learning outcomes (Egilsdottir et al. 2022:4). Nursing, in the researcher’s opinion, is a programme that requires the integration of theoretical and clinical components. In this regard, theory is taught in class, skills are demonstrated in the simulation lab and theory is put into practice in the clinical learning environment. Skills presentation at the simulation laboratory was not possible during the COVID-19 pandemic because the current curriculum was designed for traditional teaching rather than online learning, prompting the need to redesign the curriculum.

## Conclusion

The focus of this narrative literature review was the recommendations to address the online learning challenges in nursing education during the COVID-19 pandemic. The study focuses on studies published between 2020 and 2022; this is since the start of COVID-19 in December 2019, and the majority of researchers started writing and publishing articles in 2020 when the WHO declared it a pandemic on 30 January 2020. This narrative literature review identified four themes, namely provision of adequate resources, monitoring of academic dishonesty, provision of technical support and revision of the curriculum. Prior to putting any new software into the system, proper orientation and induction protocols are necessary, accompanied by unwavering support from NEIs for nurse educators and nursing students.

### Limitations

To find publications pertinent to this study, the researchers searched using the terms ‘recommendations for online learning challenges’ and ‘nursing education’. Possibly if the same study is conducted using the same concepts as ‘interventions to address online learning challenges’ and ‘digital learning’, the outcomes of the study might differ.

### Recommendations

More research is needed to develop recommendations for addressing the issues of online learning in nursing education during the COVID-19 pandemic. The NEIs should also implement mechanisms to address the problems of online learning in nursing education during the COVID-19 pandemic.
